# Linear correlation between patellar positioning and rotation of the lower limb in radiographic imaging: a 3D simulation study

**DOI:** 10.1007/s00167-023-07466-0

**Published:** 2023-06-17

**Authors:** Maximilian Jörgens, Josef Brunner, Maximilian Weigert, Markus Bormann, Elisabeth Böhm, Wolfang Böcker, Alexander C. Paulus, Denis Ehrl, Julian Fürmetz

**Affiliations:** 1 Department of Orthopaedics and Trauma Surgery, Musculoskeletal University Center Munich (MUM), University Hospital, LMU, Munich, Germany; 2 Statistical Consulting Unit StaBLab, LMU, Munich, Germany; 3 Division of Hand, Plastic and Aesthetic Surgery, University Hospital, LMU, Munich, Germany; 4 Department of Trauma Surgery, BG Unfallklinikum Murnau, Murnau, Germany

**Keywords:** Knee, Lower limb rotation, Patellar position, Long-standing radiographs, Centralized patella

## Abstract

**Purpose:**

The purpose of this study was to quantify changes in rotation of the lower limb between image pairs based on patellar position. Additionally, we investigated the differences in alignment between centralized patellar and orthograde-positioned condyles.

**Methods:**

Three-dimensional models of 30 paired legs were aligned in neutral position with condyles orthogonal to the sagittal axis and then rotated internally and externally in 1° increments up to 15°. For each rotation, the deviation of the patella and the subsequent changes in alignment parameters were calculated and plotted using a linear regression model. Differences between neutral position and patellar centralization were analysed qualitatively.

**Results:**

A linear relationship between lower limb rotation and patellar position can be postulated. The regression model (*R*^2^ = 0.99) calculated a change of the patellar position of − 0.9 mm per degree rotation and alignment parameters showed small changes due to rotation. The physiological lateralization of the patella at neutral position was on average − 8.3 mm (SD: ± 5.4 mm). From neutral position, internal rotation that led to a centralized patella was on average − 9.8° (SD: ± 5.2°).

**Conclusion:**

The approximately linear dependence of the patellar position on rotation allows an inverse estimation of the rotation during image acquisition and its influence on the alignment parameters. As there is still no absolute consensus about lower limb positioning during image acquisition, data about the impact of a centralized patella compared to an orthograde condyle positioning on alignment parameters was provided.

**Level of evidence:**

IV.

## Introduction

Long leg radiographs (LLR) remain essential for preoperative planning of open wedge tibial osteotomies (HTO) and total knee arthroplasties (TKA) as they allow standardized, simple, rapid image acquisition and highly sensitive identification of anatomical variations through reliable mechanical axis (MA) assessment [1]. Although three-dimensional (3D) imaging techniques with computed tomography (CT), magnetic resonance imaging (MRI) or digital volume tomography (DVT) are becoming increasingly important in surgical planning, they are still just performed in rare and complex cases, occasionally accompanied by patient-specific implants [3, 16].

In both deformity correction and TKA, LLRs are required not only for preoperative planning but also for postoperative examination of surgical precision, which is mandatory in some countries [6]. Unfortunately, there is no absolute consensus about the correct positioning of the lower limb during image acquisition yet. According to the initial definition of LLRs by D. Paley, LLRs should be obtained in true anterior–posterior (AP) view of the knee with the patella centred between the femoral condyles, which was the standard adjustment protocol for many years [17]. In most cases it is necessary to rotate the lower limb to achieve a position with centralized patella. Several newer studies showed that this rotation alters the alignment of the mechanical axis and various angles significantly. Consequently, all further examinations and planning procedures might be prone to error [20]. Alternatively, lower limbs should be orientated “knee forward”, with the femur condyles orthograde to the sagittal axis, parallel with the frontal reference plane, and tangential to the radiographic detector plane. This modality tends to be less influenced by present patellofemoral malalignment and tibial torsion [20].

However, the determination of angles in LLRs is highly sensible to rotational influence and prone to error, as several previous studies have demonstrated a significant decrease in medial proximal tibial angle (MPTA) and hip-knee joint angle (HKA) due to rotation [1, 2, 7]. In particular, deformities, osteoarthritis, and restricted mobility in general result in malrotation between 20° of external and internal rotation [9–11]. So far, this factor has not been considered while calculating the surgical precision between postoperative images and preoperative planning. There is already an approach to assess rotation based on tibiofibular overlap, but not yet on patellar position [12].

In this study, a linear correlation between the degree of rotation and the changes in patellar position was postulated and confirmed. It would therefore be possible to calculate alignment changes between image pairs. A further result was the clinically relevant impact on alignment parameters due to the change of focus in LLRs from true AP images with a centralized patella to knee forward images.

## Methods

Overall, 60 3D-bone models of the lower limb that were already created from existing anonymized CT data of 30 randomly selected patients (18–50 years old) were used [4, 5]. As it was aimed to cover side differences between left and right limbs, both legs of each of the 30 patients were included in the study. All models were generated from post-mortem conducted CT-data that were already evaluated in previous research projects and showed limb alignment parameters that ranked within a range of reported physiological norm values. The study was approved by the Ethics Committee of the Ludwig-Maximilians-University Munich (Nr. 17–044). In zero position, mean value for HKA angle was 180.1° (SD: ± 3.1°) and for the MPTA angle 87.7° (SD: ± 2.6°). The mean mechanical axis deviation (MAD) was measured to be 6.2 mm (SD: ± 8.4 mm), which is also within the range reported by Paley et al. as the physiological norm (10 mm ± 7 mm) [2, 17, 18]. Exclusion criteria were advanced osteoarthritis of the hip or knee joint, radiographic evidence of previous realignment surgery, fractures, and any lower extremity joint replacement. The CT scans were performed on a GE HD750 CT (GE Healthcare, Chicago, IL, USA) with standardized CT parameters (slice thickness 1.25-mmm in bone kernel, helical acquisition, 120 kV, 0.8 s/rotation, 0.984:1 pitch factor, Scan field of view (SFOV) large body, dose modulation AutomA 100–650 mA with Noise Index 8.84). Following standard procedures, the images were obtained from cranial to caudal with the patients’ legs fully extended. Digital 3D models of the legs were created using the software programs Mimics 14.0 (Materialize, Leuven, Belgium) and Geomagic Studio 2014 (3D Systems, Morrisville, NC, USA), validated rendering software for segmentation and computation, similar to 3D rendering procedures used in daily clinical practice [5]. As it was aimed to imitate LLR-images in-line with some standardized protocols, all models were aligned with the femoral epicondyles parallel to the radiographic imaging detector indicating the neutral position [20].

Overall, 37 validated and reproducible 3D landmarks on patella, femur, and tibia, which were defined and evaluated in a previous inter- and intraobserver controlled study and followed considerations of clinical practicability, were integrated into the models [5]. All landmarks were defined and evaluated by four senior orthopaedic surgeons that considered literature-based standard protocols for the implementation [4, 5]. An important characteristic of these landmarks is that they can be easily retrieved on conventional radiographs and enable adaptation of our 3D simulation on two-dimensional (2D) LLRs [5, 18]. As defined by Moreland et al. the centre of the femoral notch point was used as the femoral knee centre (FKC) and the midpoint of the tibial spines was used as the tibial knee centre (TKC) [5, 15]. The centre of the femoral head and the centre of the tibial articular surface of the ankle joint (AJC) defined the longitudinal axis [7, 18]. Further, the MAD was measured conventionally as the distance from the centre of the knee joint to the mechanical axis through the centre of the femoral head and AJC. The most medial and lateral points of the patella defined the patella medial pole (PMP) and the patella lateral pole (PLP), respectively [4]. Additionally, the most proximal (PRPP) and the most distal (PRDP) points on the patella ridge were marked for further considerations.

### Coordinate system

The models were set in a coordinate system, which enabled us to adjust the position of every leg in an approximately identical coronal and sagittal position with the femoral epicondyles parallel to the imaging detector. Compared to this zero position, we could relate position changes due to rotation.

**Fig. 1:**
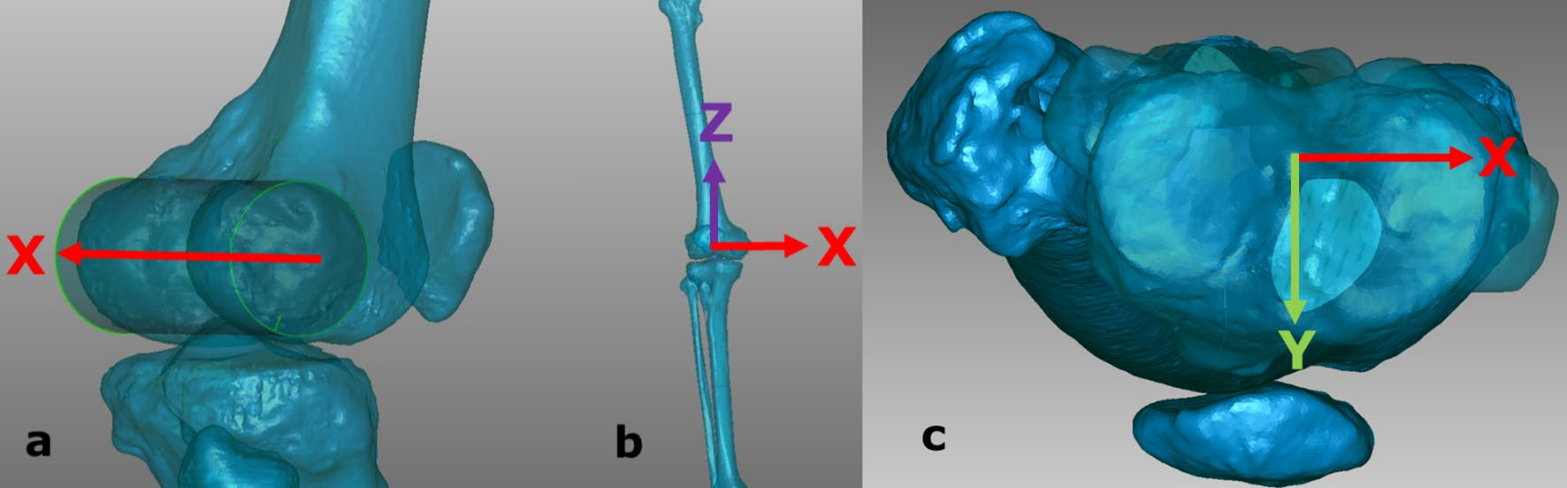
Implementation of the new coordinate system; left **a** x-axis (medial–lateral, red): “best fit” cylinder of the femoral epicondyles with the trans-epicondylar-central vector as best approximation of the knee’s flexion axis [13, 19]; middle **b** frontal view of the model with integrated coordinate system (x-axis = red, z-axis = violet); right **c** epicondylar view of the zero point of the coordinate system (x-axis = red, y-axis = green)

We first defined the medial–lateral axis by creating a geometrical “best fit” cylinder of the femoral epicondyles and using the central vector of the transepicondylar axis (Fig. 1a) [13, 19]. We hypothesized the trans-epicondylar axis to be parallel to the tangents of the femoral epicondyles and therefore suitable for defining the coronal plane of the coordinate system, as it can be seen as an imitation of a 2D-imaging detector plane [12, 22]. The sagittal and vertical alignment was defined by two perpendicular axes orthogonal to the trans-epicondylar axis through femoral notch point and the centre of the femoral head. Thus, the zero point of the coordinate system was located at the intersection of these three axes at the centre of the epicondylar cylinder (Fig. 1b, c).

All models were aligned to the new coordinate system resulting in their individual physiological neutral position. Negative measurements indicated the lower extremity to be internally rotated and positive measurements represented external rotation around the longitudinal mechanical axis [9].

### Angular measurements

The connecting lines between the centre of the femoral head and femoral notch point as well as between TKC and AJC were drawn to enable HKA angle measurement. The HKA is defined as the medial angle between those two vectors, representing the mechanical femoral axis and the mechanical tibial axis [8, 9, 18]. MAD, as the distance of the mechanical femoral axis from the centre of the knee joint, was measured by default [17, 18]. We used the most proximal lateral and medial points of the tibia to describe the medial proximal tibial angle (MPTA) [5, 18].

As we aimed to quantify the influence of lower limb rotation on patellar tracking, we defined a specific patellar position in relation to TKC and calculated the distance of both points. We imitated superimposed CT imaging by projecting the midpoint of the PLP-PMP vector and TKC onto a shared line in the same coronal and transversal plane.

### Determination of the patellar position

**Fig. 2:**
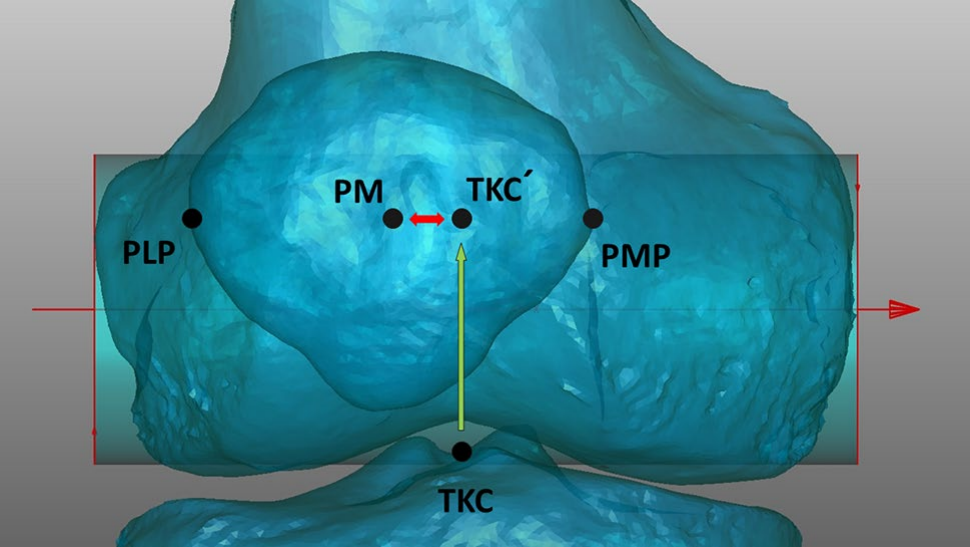
Deviation of the patellar midpoint (PM) in relation to the tibial centre of the knee (TKC) in zero position; model aligned with femoral condyles parallel to the imaging detector in zero position; PM: patellar midpoint on connecting line between outer edges of the patella defined in the frontal view; green arrow shows TKC’ as a projection of TKC on a shared projected line in the same transversal and coronal plane as PM; distance of projected points was measured (red arrow)

The following formula was used to calculate absolute values for changes of the patellar alignment (Fig. 2). The calculations were performed mathematically using an application-based interface for Python scripting.

$${\text{patellar position}}\, = \,\left[ {{\text{PLP }}\, + \,\left( {{\text{PMP}} - {\text{PLP}}} \right)/{2}} \right] \, {-} \, \left[ {{\text{TKC}}} \right]$$

PLP = Patella lateral pole (indicating the most lateral point of the patella).

PMP = Patella medial pole (indicating the most medial point of the patella).

TKC = Tibial Knee Centre (Midpoint of the medial and lateral intercondylar tubercle).

### 3D simulation of rotation

**Fig. 3:**
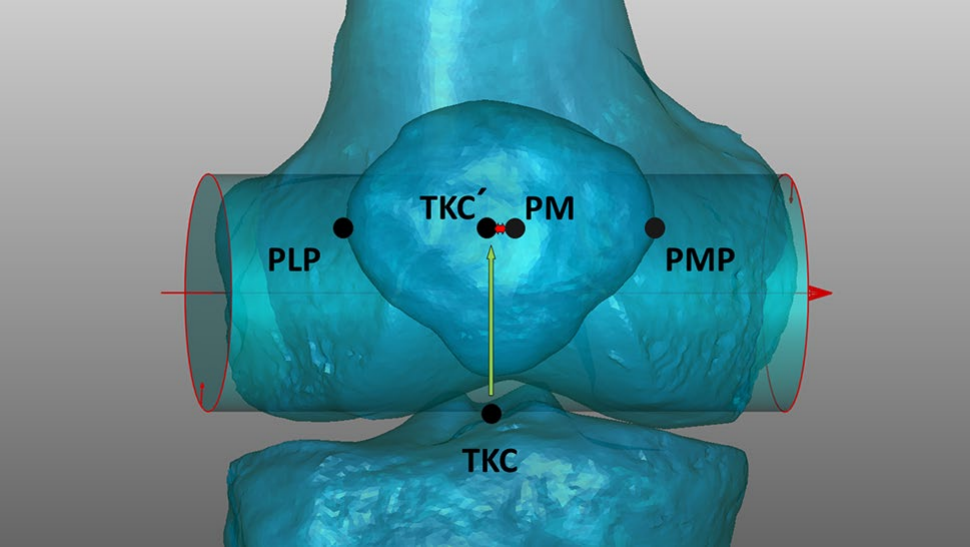
Deviation of the patellar midpoint (PM) in relation to the tibial centre of the knee (TKC) with 15° internal rotation; model aligned in 15° internal rotation; patella centralized between the femoral condyles and condyles unparallel to the imaging detector; PM almost congruent with projected (green arrow) TKC’ point; changes in alignment of the patella due to rotation was measured by the length of the projected connection between PM and TKC’ (red arrow like in Fig. 2);

For each model the alignment parameters in the neutral position with 0° of rotation were determined as the initial values for all subsequent measurements. The leg was then rotated along the longitudinal axis in 1° increments up to 15° internally (-) and 15° externally (+) and after every rotational step, the HKA, MAD, MPTA and the deviation of the patella from the zero position were measured (Fig. 3). Accordingly, for every model 31 positions of rotation and 1860 positions were obtained in total.

### Comparison of patella centralisation with neutral position

**Fig. 4:**
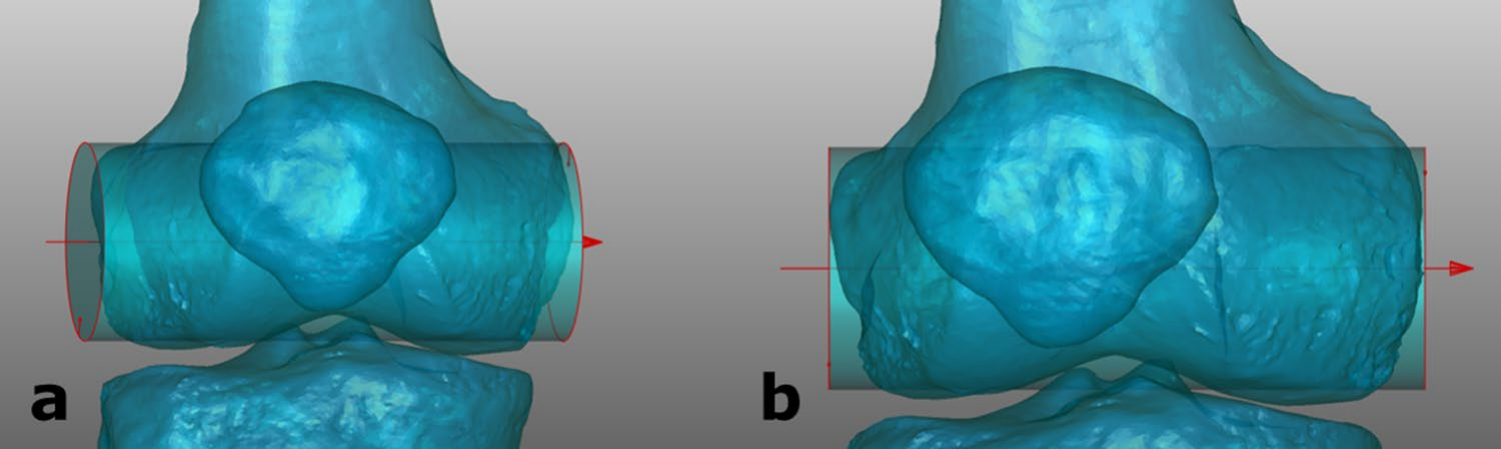
Comparison between images with focus on a centralized patella and images with condyles parallel to the imaging detector; left **a** right knee in true AP position with the patella centralized between the femoral condyles and internally rotated condyles; right **b** right knee in knee forward position with femoral condyles parallel to the imaging detector and lateralized patella

The aim was to quantify alignment changes within image pairs of one image in true AP position with a centralized patella and one knee forward image with femoral condyles parallel to the imaging detector. For every model, the rotational position with the patella centralized between the femoral condyles was compared with the zero position in knee forward orientation showing the femoral condyles parallel to the imaging detector (Fig. 4).

### Projection into the two-dimensional coronary plane

In addition to the common surveys of 3D angles, all measurements were projected into the coronal plane to mimic 2D radiographic imaging to assess the shortcomings of 2D imaging compared to 3D reality [5, 11, 14]. To generate valid angular and distance measurements, all points, angles, and distances, were calculated using a Python script to ensure an automated and standardised method.

### Statistical analysis

**Fig. 5:**
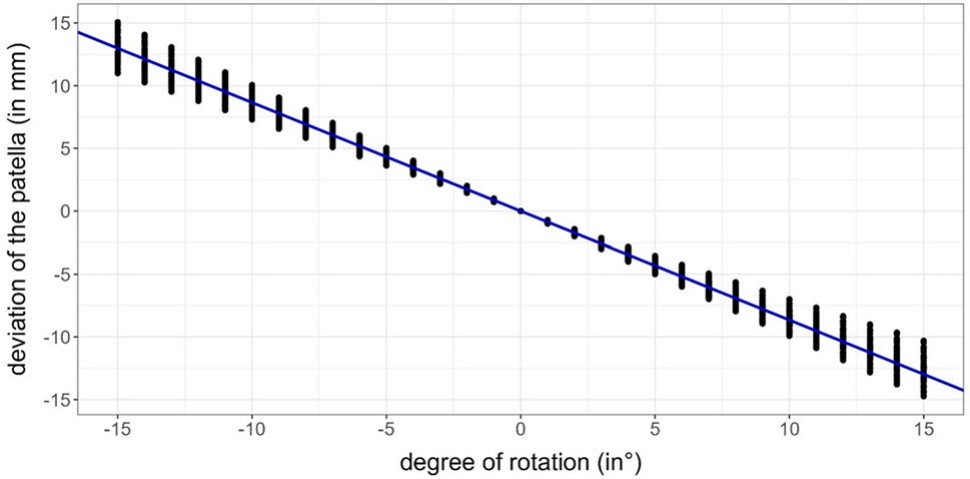
Estimated deviation of the patellar position dependent on the degree of rotation; based on calculations of a linear regression model; (negative degree (in °) of rotation = internal rotation, positive degree (in °) of rotation = external rotation); (negative values of deviation (in mm) =  lateralization; positive values of deviation (in mm) =  medialization)

Alteration of the alignment parameters compared to the neutral position with parallel condyles and a potentially decentralized patella were analysed in a qualitative manner. Correlation and regression analyses were performed to examine the association between the degree of rotation and the deviation of the patella from the zero position in an exploratory way. Linear regression models were fitted to estimate expected changes in the deviation of the patella given the degree of rotation. Due to the usage of leg-specific deviations, these models implicitly take the occurrence of repeated measurements into account. Linear mixed models were used to analyse the impact of the degree of rotation on clinical parameters MAD, HKA and MPTA, with the degree of rotation here considered as categorical variable with increments of 5° (Fig. 5) [21]. All statistical analyses were conducted using the statistical software R (R: A language and environment for statistical computing. R Foundation for Statistical Computing, Vienna, Austria; version 4.1.0).

## Results

In neutral position, mean value of the patellar position was − 8.3 mm (SD: ± 5.4 mm) externally oriented. The patella was more medialized during internal rotation and more lateralized during external rotation as it is shown in Fig. 5. Calculations of the linear regression model (*R*^2^ = 0.99) indicated a change of patellar position by − 0.9 mm per degree limb rotation. Analysing the results of the simulation, an increase in the HKA and the MAD with internal rotation and a decrease with external rotation could be seen. Conversely, the MPTA decreased with internal rotation and increased with external rotation (Table 1). The mixed linear regression model (*R*^2^ conditional = 0.99) calculated approximately a − 0.03° change of measured HKA per degree limb rotation and a 0.02° change of MPTA per degree limb rotation.

**Table 1:** Overview of deviations from zero position due to rotation for several alignment parameters derived from the (mixed) linear regression model (*n* = 60)

Deviation from zero position/degree of rotation	− 15°	− 10°	− 5°	5°	10°	15°
Patellar position (in mm)	13	8.7	4.3	− 4.3	− 8.7	− 13
HKA (in°)	0.5	0.3	0.1	− 0.1	− 0.2	− 0.3
MAD (in mm)	0.4	0.3	0.2	− 0.2	− 0.5	− 0.8
MPTA (in°)	− 0.2	− 0.2	− 0.1	0.1	0.3	0.5

*MPTA* medial proximal tibial angle, *HKA* hip knee ankle angle, *MAD* mechanical axis deviation

Most of the investigated parameters showed clinically relevant deviations due to change of orientation in model positioning from a centralized patella to parallel condyles (Table 2). Mean internal rotation that led to a centralized patella was − 9.8° (SD: ± 5.2°).

**Table 2:** Alteration of alignment parameters due to change of orientation of the model from parallel condyles to a centralized patella (*n* = 60)

Centralized patella/parallel condyles	Degree of rotation (in º)	HKA (in º)	MAD (in mm)	MPTA (in º)
Mean	− 9.8	0.2	0.7	− 0.2
SD	5.2	0.9	3.3	1.0
Max alteration internal	− 24.0	− 1.8	− 7.7	− 2.6
Max alteration external	1.0	3.1	9.1	2.4

*HKA* hip knee ankle angle, *MAD* mechanical axis deviation, *MPTA* medial proximal tibial angle

## Discussion

The most important results of this study were the clinically relevant differences of alignment between true AP images with a centralized patella and knee forward images with parallel condyles. Another interesting finding was the approximately linear relationship between the degree of lower limb rotation and the patellar position due to rotation. Taken together, these results add a parameter to those currently considered when regarding the influence of rotation on lower limb alignment.

In a previous study, Maderbacher et al. already investigated malrotation in LLRs that were conducted in “true AP” view. They found large heterogeneity of rotational positions in LLRs ranging from 30° internal to 22° external rotation comparable to the values we found in our examination [11]. They further examined underlying malrotation by assessing the projection overlap of the proximal fibula and tibia using radiographic images for calculations [12]. Similar to our study, CT scans of 50 patients in different rotation positions were analysed and a strong correlation between rotation and tibiofibular overlap was found between 20° internal and 40° external rotation. A formula for determining knee rotation in radiographs was obtained by multiregression analysis and further studies confirmed their observation [11, 12]. As we wanted to establish an easier approach to predict present knee rotation and subsequent influence on alignment parameters, we focused on relative patellar position and differences between parallel femoral condyles and centralized patella.

Lonner et al. demonstrated that 5.7° valgus at 20° of internal rotation could decrease to 2.6° at 20° of external rotation, showing considerable differences to the range Maderbacher et al. take to be the common malrotation present in LLRs [10, 11].

In their CT-based 3D simulation study, Jamali et al. also investigated the influence of rotation on alignment parameters and reported values of 5.43° to 5.08° AMA between 12° internal and 12° external rotation with an average change of 0.0146° per degree of rotation [7]. The changes of HKA with 0.03° and MPTA with 0.02° per degree of rotation were in a similar range.

This study is limited in several ways. First, the investigations were performed on healthy extended legs of a random patient cohort. Possible population-dependent factors such as weight, height, or gender could not be analysed. Knee flexion, which can occur after surgery, was also not examined. Second, patients with obvious osteoarthritis or previously known deformities of the lower extremity were excluded, even though it can be assumed that the observed effects are even stronger in this group of patients. Third, possible soft tissue or ligament structures bias the position of the patella and were not considered. Fourth, parallel X-rays were assumed, like EOS imaging or DVT, but in conventional radiographic imaging the X-ray beam is divergent. Fifth, image acquisition was done in prone position in contrast to LLRs in standing weight bearing position.

The observed combination of data provides a useful tool for clinicians to predict underlying malrotation (− 0.9 mm = 1°), when image pairs show differences in patellar position. Absent intervention on the patella explaining positional changes, orientation of the femoral condyles in reference to the imaging detector should be controlled to exclude as cause for an altered image acquisition position. As there is still no absolute consensus about the optimal positioning of the limb during image acquisition, we advise the clinician to be aware of possible alignment changes that come along with the necessary rotation of the leg to obtain images with a centralized patella.

These results allow for the easy calculation of rotationally induced changes to imaging, which are minor with the knee extended and in the absence of any relevant deformity.

## Conclusion

The approximately linear dependence of the patellar position on rotation allows an inverse estimation of the rotation during image acquisition and its influence on the alignment parameters. As there is still no absolute consensus about lower limb positioning during image acquisition, data on the impact that a centralized patella has on alignment parameters compared to that of an orthograde condyle positioning was provided in this study.

## Data Availability

The data that support the findings of this study are available from the corresponding author, MJ, upon reasonable request.
